# Supplementary material for: Characterization of a New Biofunctional, Exolytic Alginate Lyase from Tamlana sp. s12 with High Catalytic Activity and Cold-Adapted Features
Source: Mar Drugs. 2021 Mar 28;19(4):191. doi: 10.3390/md19040191 (PMC8065536; doi:10.3390/md19040191)
Supplement: Supplementary file 1 [file marinedrugs-19-00191-s001.pdf]

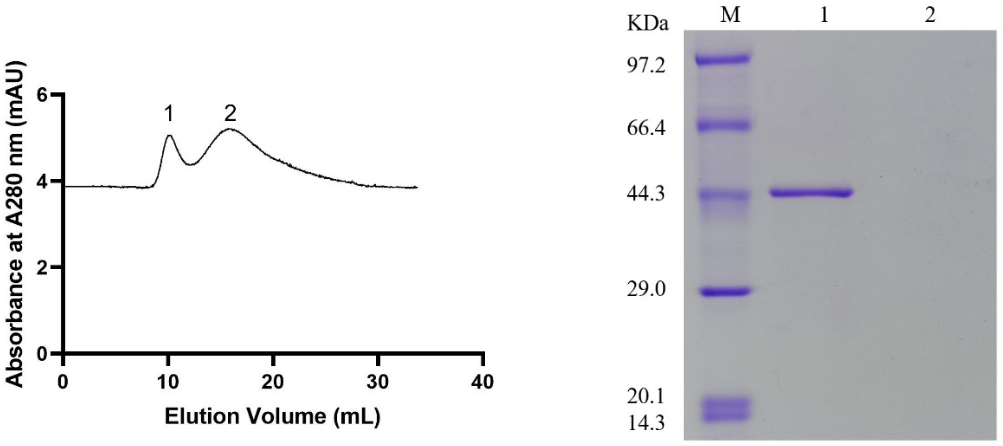

(**a**) (**b**)

Figure S1. The state of the purified rAlys1 in solution. (a) The purified rAlys1 was gel-filtered through a Superdex peptide 200 10/300 column and monitored at a wavelength of 280 nm. (b) SDS-PAGE analysis of the state of the purified rAlys1. M, protein marker; column 1, the elution of second peak; column 2, the elution of first peak.

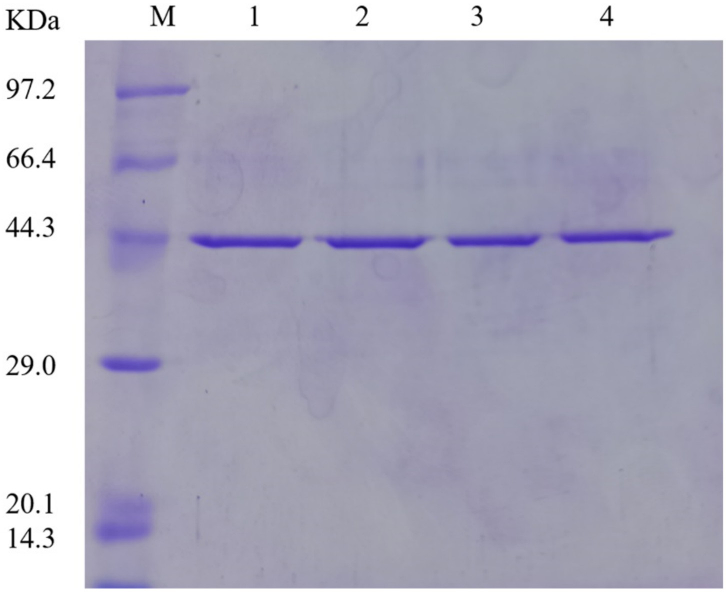

Figure S2. SDS-PAGE analysis of alginate lyases rAlys1 and its mutants.
M, protein marker; column 1, rAlys1; column 2, Q127A; column 3, H129A; column 4, Y279A.

Table S1. Primers used for mutations of rAlys1

Mutagenesis	Primers Sequences
Q127A	5'-CTTTTTCAACCGTCGTTGGAGCAATTCATAGTGATGAAGGAC-3'
	5'-GTCCTTCATCACTATGAATTGCTCCAACGACGGTTGAAAAAG-3'
H129A	5'-CCGTCGTTGGACAAATTGCTAGTGATGAAGGACACG-3'
	5'-CGTGTCCTTCATCACTAGCAATTTGTCCAACGACGG-3'
Y279A	5'-CAATATTTTAAACAAGGGGCTGCCAACCAATCGAATGG-3'
	5'-CCATTCGATTGGTTGGCAGCCCCTTGTTTAAAATATTG-3'
